# Using polygenic scores to assess liability to antisocial behavior

**DOI:** 10.18699/vjgb-25-91

**Published:** 2025-10

**Authors:** A.V. Kazantseva, D.V. Yakovleva, Yu.D. Davydova, E.K. Khusnutdinova

**Affiliations:** Institute of Biochemistry and Genetics – Subdivision of the Ufa Federal Research Centre of the Russian Academy of Sciences, Ufa, Russia; Institute of Biochemistry and Genetics – Subdivision of the Ufa Federal Research Centre of the Russian Academy of Sciences, Ufa, Russia Ufa University of Science and Technology, Ufa, Russia; Institute of Biochemistry and Genetics – Subdivision of the Ufa Federal Research Centre of the Russian Academy of Sciences, Ufa, Russia; Institute of Biochemistry and Genetics – Subdivision of the Ufa Federal Research Centre of the Russian Academy of Sciences, Ufa, Russia

**Keywords:** aggression, homicide, G × E interaction, polygenic score, regression model, ROC-analysis, social factors, агрессия, убийство, ген-средовые взаимодействия, полигенная оценка, регрессионная модель, ROC-анализ, социальные факторы

## Abstract

To date, several genome-wide association studies (GWAS) of antisocial behavior (ASB) have been conducted in Europeans, which promoted research aimed at evaluating liability to ASB-related phenotypes in independent samples. Such studies implemented a polygenic score (PGS) approach, which represents a composite score considering a number of “risky” alleles. Since no GWAS of ASB has been conducted in Russians, the present study aimed to perform a replication study of liability to severe criminal behavior (homicide) in individuals from Russia using PGS. Moreover, we sought to obtain the best model considering PGS and potential social factors as predictors. Genotyping of the “top” ten SNPs previously identified in GWAS meta-analysis of ASB (CADM2, REV3L, FOXP1, FOXP2, BDNF, FURIN, XKR6, TMEM18, SORCS3, and ZIC4 genes) was conducted via real-time PCR in 227 homicide offenders and 254 healthy donors from the Volga-Ural region of Russia. Multiple regression models included “weighted” and “unweighted” PGS and potential social factors as predictors. The best regression model of liability to severe ASB was based on genetic effects of examined SNPs and social predictors, including traumatic brain injury, severe chronic disease, and tobacco smoking, which was more pronounced among subjects with a family history of mental illness (p = 2 × 10–13). PGS alone explained a small proportion of variance in liability to ASB (1.1–1.5 %), while the inclusion of social parameters increased variance explained (16.2–21.2 %). Revealed findings evidence a higher impact of social factors than a composite effect of selected “top” SNPs in predicting liability to ASB in the examined cohort. A higher probability of ASB was linked to comorbid substance abuse, traumatic brain injury, and family history of mental illness, which may also represent a result of a “risky” genetic profile.

## Abstract (ru)

Проведенные к настоящему времени полногеномные анализы ассоциаций (GWAS) антисоциального поведения (АП) в европейских популяциях стали предпосылкой для дальнейших исследований по оценке предрасположенности к развитию схожих фенотипов в независимых выборках. В таких работах используются полигенные показатели, которые представляют собой обобщенный балл, учитывающий число «рисковых» аллелей по каждому включенному генетическому локусу. Поскольку в РФ не было проведено GWAS АП, цель настоящего исследования – проведение репликативного исследования предрасположенности к манифестации крайних форм АП (убийств) в российской когорте с использованием подсчета полигенных показателей. Кроме того, задачей было также выявление наилучшей модели, основанной на включении полигенного показателя и социальных факторов в качестве предикторов. Генотипирование 10 «топовых» SNP, идентифицированных ранее в метаанализе GWAS АП (в генах CADM2, REV3L, FOXP1, FOXP2, BDNF, FURIN, XKR6, TMEM18, SORCS3, ZIC4), проведено с помощью ПЦР в реальном времени у лиц, совершивших убийства (N = 227), и в контрольной группе (N = 254) из Волго-Уральского региона РФ. Множественный регрессионный анализ основывался на включении «взвешенных» и «невзвешенных» полигенных показателей и потенциальных социальных факторов в качестве предикторов. Наилучшая регрессионная модель предрасположенно сти к манифестации крайних форм АП содержала данные генетического профиля по 10 локусам и социальным факторам (черепно-мозговая травма, тяжелые хронические заболевания в анамнезе, табакокурение) и была наиболее значимой для лиц с семейной отягощенностью психическими расстройствами (p = 2 × 10–13). Введение только полигенного показателя в модель объясняло небольшой процент вариации в предрасположенности к АП (1.1–1.5 %), тогда как добавление социальных предикторов увеличивало процент объясненной вариации (16.2–21.2 %). Полученные результаты указывают на большую значимость социальных факторов по сравнению с кумулятивным эффектом 10 локусов в предикции развития АП в исследуемой выборке. Повышенная вероятность его манифестации связана с наличием коморбидного аддиктивного поведения, черепно-мозговой травмы и семейной отягощенности психопатологиями, что может быть результатом наличия «рискового» генетического профиля

## Introduction

Aggressive behavior (AB) and antisocial behavior (ASB) represent a destructive form of social interaction aimed at causing damage to another object and resulting in its frustration. From the evolutionary point of view, enhanced aggression was required for the survival of human groups (Baron, Richardson, 2004), thus promoting certain biological benefits. Although it is suggested that the aggression level in modern society is decreased compared with early humans, it still remains significant. To be more precise, the level of severe crimes, including homicides and intentional inflictions of severe harm, accounted for 117.3 and 567.1 thousand cases in 2022 in Russia (according to the data from the Ministry of Internal Affairs of the Russian Federation, http://www.crimestat.ru). According to the data from the World Health Organization (https://www.who.int/data), the homicide rate remains significant worldwide and was estimated at 5.8 cases per 100,000 of population in 2021 in the United States (in comparison, 6.7 cases in Russia; 0.5–4.5 cases in Europe; 5–100 cases in South American countries, and 5–20 cases in Africa).

In turn, during past years, several specifically cruel cases of murder, domestic violence, and antisocial behavior at schools have shocked Russia and the neighboring countries. However, it remains impossible to predict the occurrence of severe cruelty before the crimes have been conducted. In this regard, it seems important to determine significant factors underlying ASB, which can help to predict a higher probability of manifesting cruelty and antisocial behavior. It should be noted that ASB usually manifests in the form of certain psychiatric diseases, including oppositional defiant disorder, conduct disorder, and antisocial personality disorder (Pezzoli et al., 2025). Therefore, these phenotypes can share etiology and underlying factors.

According to previous research, the main factors predisposing to ASB or related phenotypes include biological, psychological, and environmental ones (Fritz et al., 2023). Examination of biological factors, which contribute 50 to 80 % of variance in aggression (Manchia, Fanos, 2017; Odintsova et al., 2023), is mainly focused on the analysis of genetic and epigenetic effects. Logically, genetic variants (SNPs) in the genes attributed to neurotransmitter release, reuptake, and binding (Davydova et al., 2020a; Antón-Galindo et al., 2023), oxytocin and arginine vasopressin signaling (Davydova et al., 2020b; Kazantseva et al., 2021), and others (Pezzoli et al., 2025) have been tested for their relation to individual variance in aggressive behavior. However, the results of multiple studies demonstrate inconsistent findings. Another methodological approach, i. e., genome-wide association studies (GWAS), enables to identify associated SNPs under a hypothesis-free paradigm. Although to date several GWASs of antisocial behavior have been carried out, these studies differ in the examined phenotypes frequently linked with ASB (combined phenotype of externalizing behavior (Karlsson Linnér et al., 2021), impulsivity (Deng et al., 2023), problems with selfregulation (Heilbronner et al., 2021), irritability (Mbatchou et al., 2021), risky behavior (Karlsson Linnér et al., 2019)) or age groups (children (Pappa et al., 2016), adults (Tielbeek et al., 2017)). Moreover, summarized findings from ~ 1.5 million subjects identified more than 500 SNPs related to liability to externalizing behavior, including antisocial behavior, attention- deficit/hyperactivity disorder (ADHD), and addiction in a European cohort (Karlsson Linnér et al., 2021).

One of the possible applications of GWAS findings is to use them for the calculation of polygenic scores (PGS) on the basis of effect estimates obtained for each SNP in the training sample. In turn, inclusion of PGS in mathematical models can gain prediction of enhanced risk of certain complex phenotypes. To date, several attempts seeking to replicate GWAS findings in an independent sample using PGS from ASB phenotype have been made (Karlsson Linnér et al., 2021; Li et al., 2023; Tesli et al., 2024; Acland et al., 2025), which succeeded in determining some proportion of variance in liability to conduct disorder, substance use disorders, smoking, ADHD, criminal behavior, depression, posttraumatic stress disorder, unemployment, and suicidal attempts. One of the possible limitations of using PGS for predicting ASB is the ethnic origin of the examined population, since differences in allele and genotype frequencies between ethnic groups can change SNPs’ effect (Kazantseva et al., 2016). To date, no GWAS of liability to homicidal conduct has been carried out in subjects from Russia. Therefore, it is relevant to check if it is applicable to use the effect estimates obtained from combined ASB phenotype and different ethnic groups to predict the probability of conducting severe ASB in the Russian cohort.

Undoubtedly, specific environmental/social factors acting at various stages of ontogenesis affect genes’ activity via epigenetic changes in regulation of genes responsible for manifesting aggression (Borinskaya et al., 2021). In this context, the analysis of potential social factors together with genetic effects (PGS) can help to increase the prognostic significance of the final model. In addition, it is established that ASB is highly accumulated in certain groups, including subjects with comorbid mental disorders (Ip et al., 2021; Wang et al., 2024; Pezzoli et al., 2025), family history of mental illness (Han et al., 2024), addiction (Karlsson Linnér et al., 2021; Antón-Galindo et al., 2023), and unfavorable rearing conditions (Burt, 2022).

Considering the existing findings of ASB meta-analysis of European populations (Karlsson Linnér et al., 2021) and absent GWAS data for individuals from Russia, the present study aimed to evaluate the applicability of calculated polygenic scores based on existing GWAS data to predict severe ASB (homicide) in the Russian cohort. Moreover, to enhance the prognostic ability of regression models, we sought to obtain the best model with the optimal sensitivity and specificity, which assumes PGS and potential social factors as predictors

## Materials and methods

The study sample comprised 227 criminal offenders who conducted homicide and were directed to a forensic examination of present mental disorders in the Republican Clinical Psychiatric Hospital (Ufa, Russia). Only individuals without mental illness who were proven to be sane by the Court were included in the study. The examined sample consisted mainly of men (93 %) with a mean age of 41.5 ± 14.5 years. Ethnic content of the sample was the following: 48 % Russians, 34.8 % Tatars, and 17.2 % Bashkirs. The data on the social/ clinical background of enrolled subjects were obtained via a survey and included the information on present and past tobacco smoking, alcohol/opiate abuse, family history of mental illness or criminal behavior, suicidal attempts, level of education, maltreatment in childhood, severe chronic disease in anamnesis, and type of ASB (proactive or reactive aggression).

The control group was selected on the basis of correspondence to the group of criminal offenders by age, ethnicity, and gender. In total, we examined DNA samples obtained from 254 individuals who reported no family history of mental illness and were non-registered in the psychiatric database of the Republic of Bashkortostan. The study was approved by the local bioethical committee at the Institute of Biochemistry and Genetics – Subdivision of the Ufa Federal Research Centre of the Russian Academy of Sciences (Ufa, Russia) (protocol code 15, date of approval, October 12, 2017) in accordance with the 1964 Helsinki Declaration and its later amendments or comparable ethical standards.

SNP selection for PGS calculation from GWAS meta-analysis of ASB (Karlsson Linnér et al., 2021) was based on the following criteria: the lowest level of significance ( p <10–18); selection of a single SNP from a set of proxy SNPs; minor allele frequency (MAF) above 0.05 in Europeans (based on 1000 Genomes); and known regulatory effect of the SNP based on the RDB (Regulome Database, https://regulomedb. org/regulome-search) and CADD (Combined Annotation Dependent Depletion, https://cadd.gs.washington.edu) databases. The final list of selected SNPs included CADM2 rs993137, REV3L rs458806, FOXP1 rs11720703, FOXP2 rs1476535, BDNF rs6265, FURIN rs4702, XKR6 rs4240671, TMEM18 rs6711254, SORCS3 rs11596214, and ZIC4 rs2279829, which were used for PGS calculation, and is reported in Table 1. Genotyping of previously extracted DNA of the control group and criminal offenders was carried out using real-time PCR with KASP chemistry (LGC Genomics, UK).

**Table 1.**
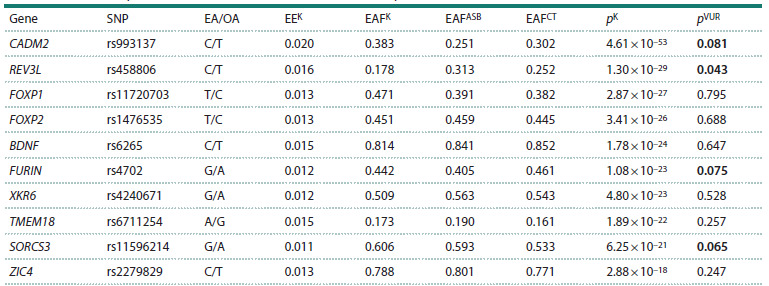
Examined top SNPs linked to antisocial behavior: data from previous ASB GWAS and the VUR cohort

All examined SNPs corresponded to the Hardy–Weinberg equilibrium ( p > 0.05). At the second stage, we calculated PGS based on effect estimates obtained from R. Karlsson Linnér et al. (2021). Namely, PGS for each individual from our sample was calculated on the basis of inclusion of 1) SNPs under p < 0.1 (“weighted” effect), 2) all SNPs (using “weighted” effect), 3) all SNPs (using “unweighted” effect). Calculation of “weighted” and “unweighted” PGS was previously explained in detail (Kazantseva et al., 2023a). Briefly, individual PGS was calculated as the weighted/unweighted sum of the number of effect alleles at a certain SNP multiplied by the effect estimate (PLINK v.1.09).

Subsequently, a series of multiple logistic regressions was performed to obtain models that can predict liability to ASB in the total groups of homicide offenders, as well as in subgroups of subjects with proactive forms of aggression, comorbid substance use, or known family history of mental illness or criminal behavior. Initially, only PGS as a predictor was included, which was followed by a backward selection procedure to obtain a list of statistically significant social parameters to be included as predictors together with PGS (R v.4.4.2). To select the best predicting model, we have compared data on the lowest p-value, the highest proportion of variance (Nagelkerke pseudo-R2) explaining liability to ASB, and the highest area under the ROC curve (AUC) for each model.

## Results

At the initial stage of the study, we examined the presence of significant differences between the criminal offenders and the control group in the examined social factors (Table 2). We have observed the differences in the proportion of individuals characterized by severe somatic diseases and traumatic brain injuries in anamnesis ( p = 1.2 × 10–12), depending on education level ( p = 4.5 × 10–16) and present smoking ( p = 4.0 × 10–7) between the groups.

**Table 2.**
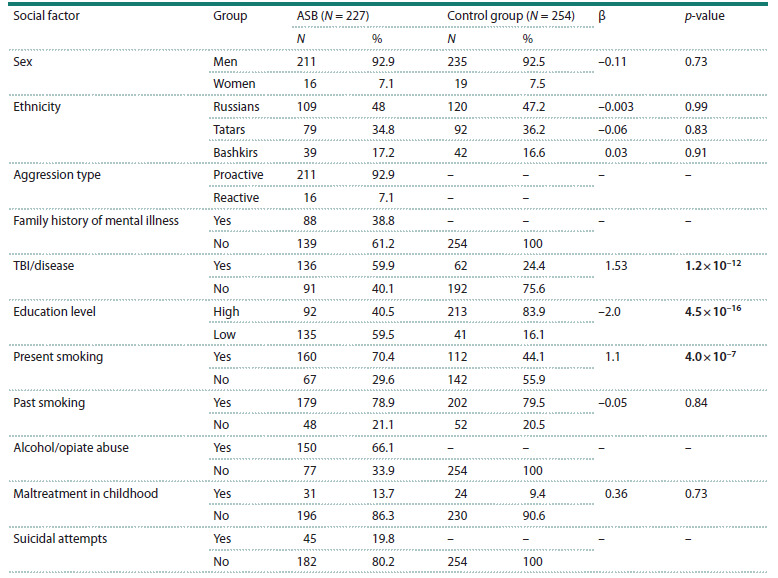
Characteristics of the examined groups of criminal offenders and healthy donors and analysis of differences in social factors between the groups Note. Statistically significant differences between the groups based on p-value < 0.05 are shown in bold. TBI/disease – traumatic brain injury or severe chronic disease in anamnesis. Dashes indicate non-applicable data.

For the genetic part of the present study we selected the “top” ten SNPs ( p < 2.9 × 10–18) identified in the previous meta-analysis GWAS of ASB (Karlsson Linnér et al., 2021). Effect estimates for alleles used for calculation of “weighted PGS” as well as effect allele frequencies in the VUR sample are given in Table 1. In addition, we have tested for statistically significant differences in allele frequencies of examined SNPs between criminal offenders and the control group in the examined cohort from the VUR, which enabled us to confirm a coincidence of four SNPs, although at a trend level ( p < 0.1): CADM2 rs993137, REV3L rs458806, FURIN rs4702, and SORCS3 rs11596214.

**Fig. 1.**
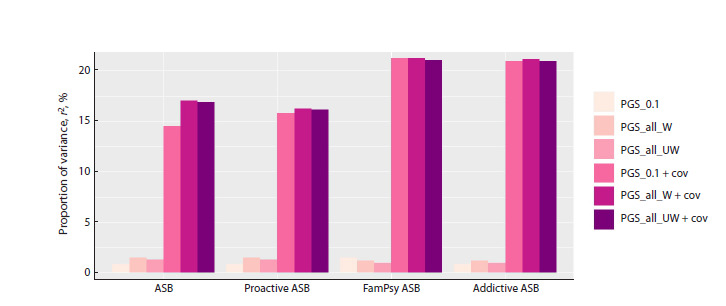
Proportion of variance (adjusted r2) in liability to antisocial behavior in the examined cohort explained by predictors included in multiple regression models based on PGS calculation of SNPs with p < 0.1 (PGS_0.1), “weighted effects” of all SNPs (PGS_all_W), “unweighted effects” of all SNPs (PGS_all_UW) with inclusion of social predictors (PGS_0.1 + cov, PGS_all_W + cov, PGS_all_UW + cov). Examined groups of ASB: total group of homicide offenders (ASB); homicide offenders with a proactive type of ASB (Proactive ASB), family history of mental illness or criminal behavior (FamPsy ASB), or substance abuse (Addictive ASB).

Primary logistic regression models that included PGS (based on four SNPs) revealed a small proportion of variance in liability to antisocial behavior in the total group (r2 = 0.9 %, p = 0.014), among subjects with a proactive form of aggression (r2 = 0.9 %, p = 0.017), with comorbid substance abuse (r2 = 0.9 %, p = 0.027), and with a family history of mental illness (r2 = 1.5 %, p = 0.014) (Table 3, Fig. 1). At the initial stage of regression analysis, we have included all social factors, including sex and ethnicity as covariates, together with PGS.

**Table 3.**
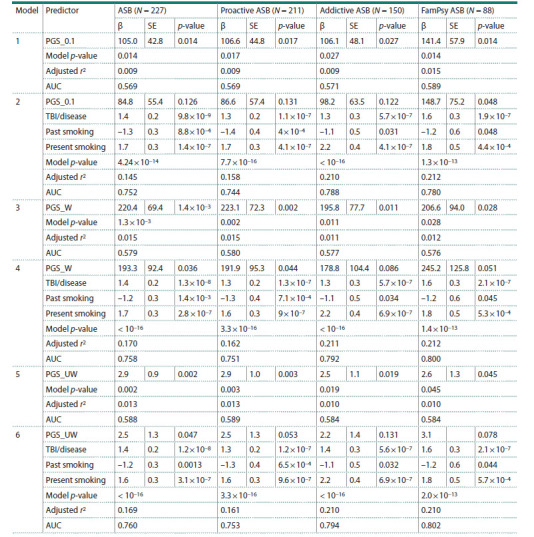
Regression models of liability to ASB based on a polygenic score and social factors as predictors Note. ASB – antisocial behavior; FamPsy ASB – ASB in individuals with a family history of mental illness; β – regression coefficient for each predictor in the model; SE – standard error of β; AUC – area under curve; TBI/disease – traumatic brain injury or severe chronic disease in anamnesis. PGS_0.1 was based on effect estimates for REV3L rs458806, FOXP1 rs11720703, XKR6 rs4240671, and SORCS3 rs11596214; PGS_W and PGS_UW were PGS based on “weighted” and “unweighted” effect estimates for ten SNPs, correspondingly.

As expected, inclusion of potential social parameters as predictors enabled an increase in the statistical significance of the models, which resulted in 14.5 % (ASB), 15.8 % (proactive ASB), 21.0 % (ASB with comorbid addiction), and 21.2 % of variance (ASB with family history of mental illness) being explained. It should be mentioned that valuable social factors comprised of traumatic brain injury (TBI) or severe chronic disease in anamnesis (β = 1.4, p = 9.8 × 10–9) and present smoking (β = 1.7, p = 1.4 × 10–7) were associated with enhanced liability to aggression, while past smoking demonstrated a positive effect on ASB decrease (β = –1.3, p = 8.8 × 10–4). The impact of other social factors together with sex and ethnicity remained insignificant after the backward selection procedure. Therefore, inclusion of the mentioned social parameters allowed us to explain up to 16.1 % of variance in developing ASB. According to determined models, we can conclude that they possess the highest prediction ability for developing ASB in individuals who have relatives with mental disorders or criminal behavior (AUC = 0.780) or have alcohol/opiate addiction (AUC = 0.788) (Table 3).

At the second stage of our analysis, we calculated PGS based on effect estimates for all examined SNPs, even if they were non-significant in the VUR sample (Table 1). Therefore regression models, which implemented “weighted” (PGS_W) and “unweighted” (PGS_UW) PGS, slightly enhanced the proportion of variance in liability to ASB compared to previous models 1 and 2 (Table 2). Namely, a combined effect of ten genetic variants explained 1.1–1.5 % (“weighted effect”) and 1.0–1.3 % (“unweighted effect”) of variance in predisposition to homicide violence. Previously mentioned social predictors remained significant and, together with PGS, enabled to enhance the proportion of variance explained (16.2–21.2 % in “weighted” PGS, 16.1–21.0 % in “unweighted” PGS).

**Fig. 2.**
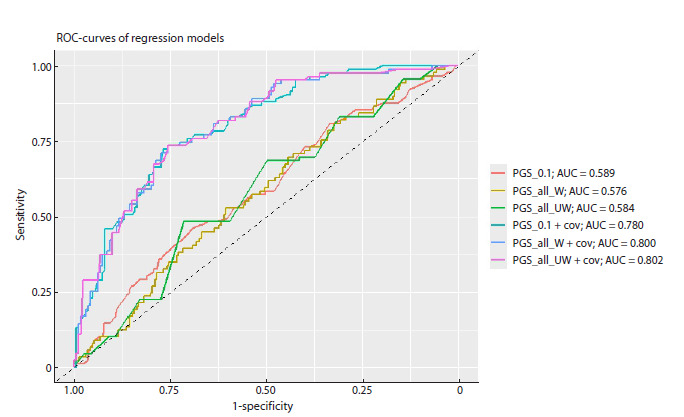
ROC curves and areas under the curves (AUCs) for various regression models predicting liability to manifest ASB in individuals with a family history of mental illness or criminal behavior (abbreviations are given as in Fig. 1).

However, it seems that the inclusion of a larger number of non-significant SNPs had a very small effect on improving the predicting abilities of the models. Nevertheless, models with ten vs. four SNPs in PGS demonstrated slightly higher prognostic ability for ASB in the total sample and in individuals with a proactive form of aggressive behavior or comorbid substance abuse (Table 2, Models 4, 6). We have also constructed ROC curves and calculated comparative areas under the curves (AUCs) for all analyzed models (Fig. 2). Finally, our findings indicate that the best regression model has higher prognostic ability (r2 = 21 %) and a moderate measure of classifier performance (AUC = 0.802) to designate subjects at high risk for developing ASB if they have family history of mental disorders.

## Discussion

In the present study, we tested different logistic regression models, which were based on calculated polygenic scores, aimed at predicting liability to homicide in individuals from the Volga-Ural region of Russia. Based on our findings, the highest prediction ability for developing ASB was observed for individuals with a family history of mental disorders and those with substance abuse comorbidity. The data revealed are not surprising, since externalizing pathology is frequently accumulated in families (Acheson et al., 2018; Han et al., 2024) due to shared genetic profiles between biological parents and offspring. On the other hand, it was reported that the same genes/genetic variants were linked to different psychiatric conditions, addiction, and antisocial behavior (Ip et al., 2021; Antón-Galindo et al., 2023; Li et al., 2023; Wang et al., 2024; Pezzoli et al., 2025), which can be explained by genes’ pleiotropy in various complex traits (Watanabe et al., 2019).

Since no significant difference in predicting ASB risk in the VUR sample was observed among models based on “weighted” and “unweighted” effects of SNPs, it can be concluded that effect estimates from GWAS of Europeans seem to be inappropriate for individuals from Russia. Therefore, future research should be focused on conducting GWAS of ASB in a Russian cohort followed by verification in the same-ethnicity independent sample. Published studies, which sought to replicate findings obtained for different populations, succeeded in using PGS from ASB to predict liability to externalizing behavior in both Europeans and African Americans (Brislin et al., 2024), although representing a small cumulative effect of genetic variants.

Our findings indicate a very small impact of selected SNPs on predicting ASB, which was based on the effect estimates from the study of summarized phenotype of externalizing pathology. The data obtained support previous findings on the small effect (0.1–4.0 %) of analyzed genetic variants (even at a genome-wide level) as polygenic scores on predicting ASB (Tielbeek et al., 2017, 2022; Tesli et al., 2024). Our previous research also revealed a small proportion of variance explained in aggression level in a general population of Russia, which was attributed to the combined effect of 30 genetic variants (Kazantseva et al., 2023b).

It is known that environmental factors play a modulating role in establishing specific patterns of behavior (Kazantseva et al., 2014), including ASB-related ones. In particular, harsh parenting (Burt, 2022), school violence (Acland et al., 2025), and affiliation with delinquent peers (Schwartz et al., 2019) were assumed to increase a risk for manifesting ASB. Regression models designed in the present study also point to a more pronounced effect of environmental factors in establishing ASB than that of the genetic component. These findings are at odds with existing studies, which also depicted valuable impact of such social factors as community violence (Musci et al., 2019), harsh parenting (Acland et al., 2025), and low parental education level (Barnes et al., 2019) under gene-byenvironment interactions

In the present study, we have observed a significant effect of present smoking and history of traumatic brain injury/severe chronic disorders on manifesting criminal behavior. One of the probable links between smoking and ASB is attributed to the influence of nicotine on the CNS via exaggerated stress sensitivity (Weltens et al., 2021) and changed in epigenetic regulation (Gould et al., 2023). It should be noted that the usually accepted environmental effects can also be due to the impact of certain genetic and epigenetic profiles, which are inherited (McAdams et al., 2013). In this regard, present smoking may represent the result of activity of the genes responsible for developing addiction and externalizing behavior. Moreover, the negative effect of smoking promoting the development of ASB later in life was only evident for individuals with predisposing genetic patterns. Namely, individuals who were subjected to prenatal smoking exposure (their mothers smoked during pregnancy) demonstrated an enhanced risk of ASB only if they were genetically related to their mothers. At the same time, no link between maternal smoking and offspring’ ASB was observed if children were developed from a donated egg (van Goozen et al., 2022).

Another significant factor affecting liability to ASB in our cohort is traumatic brain injury in anamnesis, which confirms previous data (Ryan et al., 2021; Theadom et al., 2024). It was suggested that TBI can cause abnormal morphometry of the central executive network in the brain, which can result in worsening of executive functions (Ryan et al., 2021) or exacerbate other valuable triggers, including social deprivation (Guskiewicz et al., 2003), thus promoting ASB. In summary, reported findings on the higher effect of social factors on developing ASB in the Russian cohort can probably capture the effect of other genes on the occurrence of such an “environment”.

Future research should integrate various methodological approaches, including those measuring brain activity and connectivity underlying specificity of individual behavioral responses, and consider the impact of genetic and environmental factors. For instance, there is some evidence of a link between amygdala hyper-reactivity and increased impulsivity and reduced self-regulation as a response to threatening stimuli (Dotterer et al., 2017). Another study identified a link between diminished P3(P300) amplitude of electrical potential, which was obtained as a response to a visual oddball task, and manifestation of externalizing phenotypes (Iacono, 2018; Brislin et al., 2024).

The present study has several limitations. First, the set of SNPs used for PGS calculation is rather small, which can mirror the low proportion of explained variance in liability to ASB attributed to genetic impact. To be more precise, calculated PGS in the previous meta-analysis (Karlsson Linnér et al., 2021) enabled to explain 3–4 % of variance in manifesting a combined phenotype of antisocial behavior when PGS was estimated on genetic data from 579 SNPs at the genome-wide significance level.

In turn, the present study has been focused on biallelic polymorphisms only, while other structural variations in the genome, such as tandem repeats and microdeletions/duplications, which can also contribute to genetically caused manifestations of aggression, remained unstudied within the present research. Although the examined sample represents a specific cohort of individuals with a severe form of aggressive behavior (homicide), the sample size is small, which can result in type I and II errors and requires future enlargement of the examined sample. Moreover, the obtained PGS models are limited to a number of analyzed social factors while other probably relevant factors including child-parent relationship, belonging to a criminal organization, physical or sexual violence, social isolation, personality type, etc. were non-examined. Finally, since the majority of enrolled offenders were characterized by excessive alcohol/opiate use, we cannot rule out whether the reported findings are attributed to present heavy alcohol drinking.

## Conclusion

In summary, the present study represents an attempt to create a prognostic model for developing antisocial behavior in a Russian cohort based on genetic data reported for European populations. Revealed findings present evidence for a higher impact of social factors rather than a composite effect of the selected “top” SNPs in predicting liability to ASB. Nevertheless, the best model was able to explain up to 21.2 % of variance in liability to ASB, especially in subjects with a family history of mental illness or criminal behavior, which was based on the genetic profile of ten SNPs and such social parameters as traumatic brain injury, severe chronic disease in anamnesis, and tobacco smoking. Future research in this field has to be focused on performing GWAS in a Russian cohort of criminal offenders (or persons with other types of antisocial behavior) to identify genetic loci and their effect estimates specific to the main ethnic groups from Russia. Obviously, such analyses will enable the design of models of liability to manifest ASB with higher prediction probabilities.

## Conflict of interest

The authors declare no conflict of interest.
